# Alpha-180 spin-echo-based line-scanning method for high-resolution laminar-specific fMRI in animals

**DOI:** 10.1162/imag_a_00120

**Published:** 2024-03-28

**Authors:** Sangcheon Choi, David Hike, Rolf Pohmann, Nikolai Avdievich, Lidia Gomez-Cid, Weitao Man, Klaus Scheffler, Xin Yu

**Affiliations:** Athinoula A. Martinos Center for Biomedical Imaging, Department of Radiology, Harvard Medical School, Massachusetts General Hospital, Charlestown, MA, United States; Max Planck Institute for Biological Cybernetics, Tuebingen, Baden-Wuerttemberg, Germany; Department of Biomedical Magnetic Resonance, University of Tuebingen, Tuebingen, Baden-Wuerttemberg, Germany

**Keywords:** microvascular sensitivity, laminar specificity, line-scanning, high-resolution fMRI, spin-echo

## Abstract

Laminar-specific functional magnetic resonance imaging (fMRI) has been widelyused to study circuit-specific neuronal activity by mapping spatiotemporal fMRIresponse patterns across cortical layers. Hemodynamic responses reflect indirectneuronal activity given the limitation of spatial and temporal resolution.Previously, a gradient-echo-based line-scanning fMRI (GELINE) method wasproposed with high temporal (50 ms) and spatial (50 µm) resolution tobetter characterize the fMRI onset time across cortical layers by employing twosaturation RF pulses. However, the imperfect RF saturation performance led topoor boundary definition of the reduced region of interest (ROI) and aliasingproblems outside of the ROI. Here, we propose an α (alpha)-180spin-echo-based line-scanning fMRI (SELINE) method in animals to resolve thisissue by employing a refocusing 180˚ RF pulse perpendicular to theexcitation slice (without any saturation RF pulse) and also achieve highspatiotemporal resolution. In contrast to GELINE signals which peaked at thesuperficial layer, we detected varied peaks of laminar-specific BOLD signalsacross deeper cortical layers using the SELINE method, indicating thewell-defined exclusion of the large draining-vein effect using the spin-echosequence. Furthermore, we applied the SELINE method with a 200 ms repetitiontime (TR) to sample the fast hemodynamic changes across cortical layers with aless draining vein effect. In summary, this SELINE method provides a novelacquisition scheme to identify microvascular-sensitive laminar-specific BOLDresponses across cortical depth.

## 1 Introduction

Line-scanning fMRI has been successfully applied to investigate circuit-specificneuronal activity by measuring dynamic hemodynamic responses across cortical layerswith high spatiotemporal resolution (Albers et al.,2018;S. Choi, Chen, et al., 2023;S. Choi et al., 2018;S. Choi, Xie, et al., 2023;S. Choi, Yu, et al., 2022;S. Choi, Zeng, et al., 2022;Raimondo, Priovoulos, et al., 2023;Raimondo et al., 2021;X.Yu et al., 2014). This is initially originated from Mansfield’sline-profile mapping studies in early 1970s (Mansfield & Maudsley, 1976;Mansfield et al., 1976). The advantage of the current line-scanning fMRImethod is to sample cortical layers with ultra-high spatial resolution. Meanwhile,the line-scanning method only acquires a single k-space line per timepoint, enablingan ultrafast sampling rate. This high spatiotemporal laminar fMRI sampling schemehas been being utilized for bottom-up and top-down blood-oxygenation-level-dependent(BOLD) fMRI mappings in both animal and human fMRI studies. Previously,X. Yu et al. (2014)developed a line-scanningfMRI method to delineate laminar fMRI onset time with distinct laminar-specificneural inputs such as thalamocortical input and corticocortical input in the ratbrain with high spatial (50 μm) and temporal resolution (50 ms).Line-scanning fMRI has also been combined with optogenetic control to furtherinvestigate the temporal features of the fast neural inputs across cortical layersin rodents (Albers et al., 2018). Beyondpreclinical fMRI studies, line-scanning fMRI for human brain mapping hasdemonstrated a good correspondence with BOLD responses of 2D echo planar imaging(EPI) at the same temporal scale (200 ms) (Raimondoet al., 2021). This line-scanning fMRI also motivated the corticaldepth-dependent diffusion-based fMRI mapping schemes (Balasubramanian et al., 2021). Lately, the ultra-fast line-scanning fMRIwith k-t space reshuffling scheme has even provoked some interesting investigationsof direct neuronal activity measurements (Tan Toi etal., 2022), albeit it has not been replicated in animals and humans(S.-H. Choi et al., 2023;Hodono et al., 2023).

The typical gradient echo (GRE)-based line-scanning fMRI (GELINE) method needs todampen signals outside of the region of interest (ROI) to avoid aliasing artifactsalong the phase encoding direction (X. Yu et al.,2014;Albers et al., 2018;Raimondo et al., 2021;S. Choi, Zeng, et al., 2022;Raimondo et al., 2023;S.Choi, Chen, et al., 2023;S. Choi, Xie,et al., 2023). Two saturation slices with additional RF exposure areapplied for this purpose. However, two issues should be further investigated. One isthe imperfect elimination of the aliasing artifacts (including inflow effects) dueto imperfect RF performance and inhomogeneous B0 field. The other is the specificabsorption rate (SAR) problem stemming from high duty cycle sequences. To alleviatelarge draining vein contribution to non-specific GELINE responses, large-tip-anglespin-echo based line-scanning fMRI method was previously proposed fordiffusion-based fMRI (dfMRI) studies in animals by combining both a typicalspin-echo sequence and saturation RF pulses at a short TR (Nunes et al., 2021). This study provided the first directlink between ultra-fast dfMRI signals upon forepaw stimulation and intrinsic opticalsignals upon optogenetic stimulation while demonstrating ultrafast dfMRI couldreflect neuromorphological coupling. Even though the concept of the proposedspin-echo line-scanning fMRI method was implemented in humans, laminar-specific fMRIsignals were not observed possibly due to low temporal signal-to-noise ratio (tSNR),potential motion artifacts (no motion correction applied), and/or low functionalsensitivity (Raimondo, Heij, et al., 2023).Based on our initial line-scanning fMRI study (S.Choi et al., 2018), here, we further developed an α (alpha)-180line-scanning fMRI method in animals to solve these problems while achieving highspatiotemporal resolution. We modified a spin-echo (SE) sequence by altering therefocusing 180° RF pulse perpendicular to the excitation slice (S. Choi et al., 2018;Mansfield et al., 1976;Mansfield & Maudsley, 1976). This adjustment allows to onlyhighlight a specific line-profile across the cortical layers without the need foradditional saturation RF pulses. Nevertheless, there is an inevitable trade-offbetween T2*-weighted GELINE and T2-weighted SELINE: GELINE has lowspecificity but high sensitivity to BOLD whereas SELINE has low sensitivity but highspecificity to BOLD. As reported in previous works (Boxerman et al., 1995;Budde et al.,2014;Han et al., 2019;Norris, 2012;Siero et al., 2013;Zhao et al.,2004), GRE-based BOLD responses are dominated by the macrovasculature(e.g., large draining veins) and SE-based BOLD responses have greater microvascularsensitivity (e.g., capillary vessels) than GRE-based BOLD responses, specificallyindicating extravascular and intravascular contributions to BOLD responses should betaken into account. In high-field MRI, extravascular signal changes likelypredominate while intravascular signal changes mostly diminish (Boxerman et al., 1995). In contrast to the GELINE method,the SE-based line-scanning fMRI (SELINE) method thus has the potential toeffectively exclude the surface draining vein effects. However, it should be notedthat the laminar patterns of BOLD signals in SELINE can still be highly variedacross different cortical layers in anesthetized rats. Furthermore, we can alsoshorten the repetition time (TR) to 200 ms for the SELINE method to sample thehigh-resolution T2-weighted fMRI signals, demonstrating the feasibility of the fastsampling of laminar fMRI with effective ROI selectivity in rodents.

## 2 Methods

### 2.1 Animal preparation

The study was performed in accordance with the German Animal Welfare Act(TierSchG) and Animal Welfare Laboratory Animal Ordinance (TierSchVersV). Thisis in full compliance with the guidelines of the EU Directive on the protectionof animals used for scientific purposes (2010/63/EU) and the MGH Guide for theCare and Use of Laboratory Animals. The study was reviewed by the ethicscommission (§15 TierSchG) and approved by the state authority(Regierungspräsidium, Tübingen, Baden-Württemberg, Germany)and the MGH Institutional Animal Care and Use Committee (Charlestown, MA, USA).A 12-12 h on/off lighting cycle was maintained to assure undisturbed circadianrhythm. Food and water were available ad libitum. A total of four maleSprague–Dawley rats were used in this study.

Anesthesia was first induced in the animal with 5% isoflurane in the chamber. Theanesthetized rat was intubated using a tracheal tube, and a mechanicalventilator (SAR-830, CWE, USA) was used to ventilate animals throughout thewhole experiment. Femoral arterial and venous catheterization was performed withpolyethylene tubing for blood sampling, drug administration, and constant bloodpressure measurements. After the surgery, isoflurane was switched off, and abolus of the anesthetic alpha-chloralose (80 mg/kg) was infused intravenously.After the animal was transferred to the MRI scanner, a mixture ofalpha-chloralose (26.5 mg/kg/h) and pancuronium (2 mg/kg/h) was constantlyinfused to maintain the anesthesia and reduce motion artifacts.

### 2.2 EPI fMRI acquisition

**Fig. 1.**
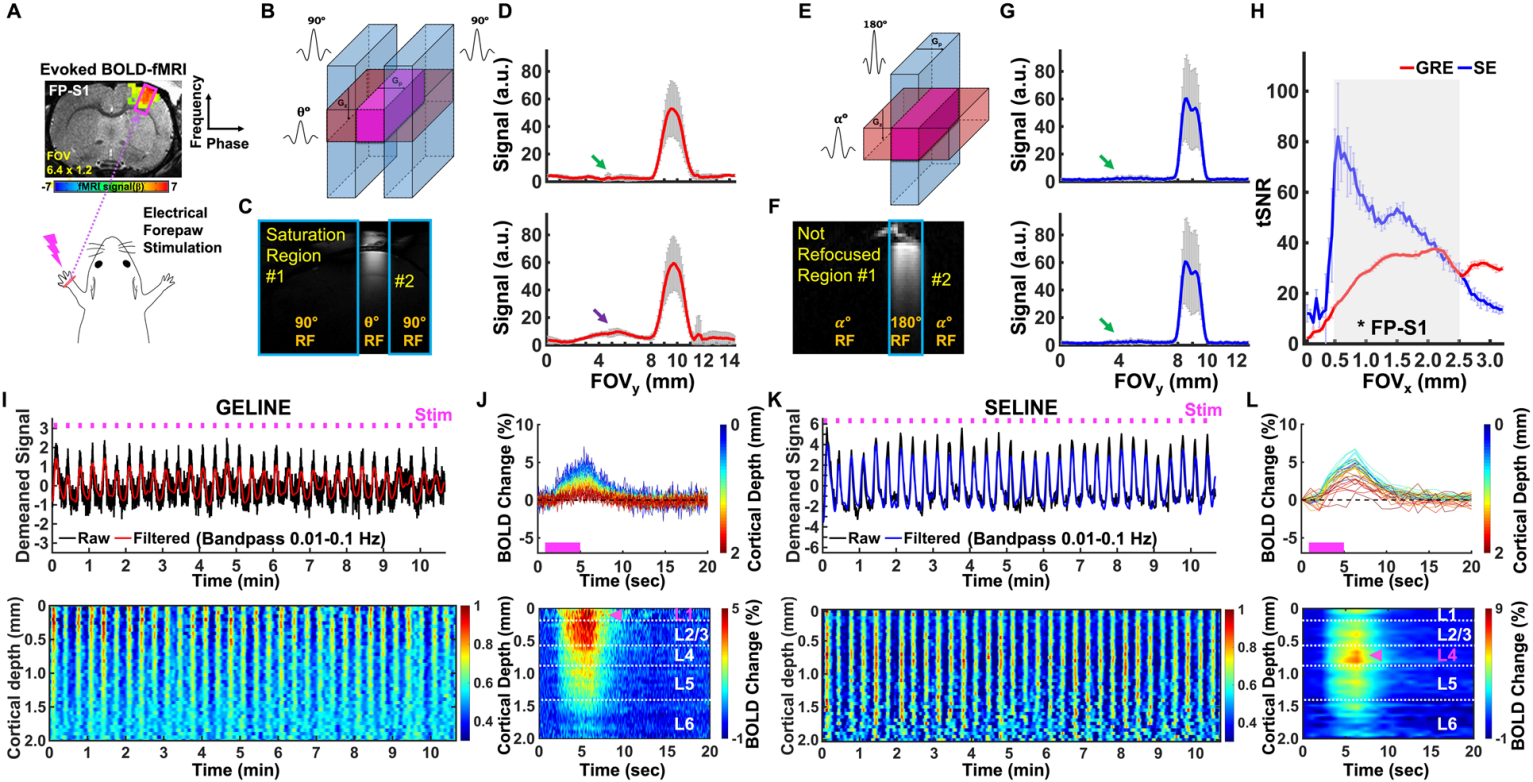
Evoked BOLD responses upon left forepaw stimulation using the GELINE andSELINE methods. (A) Schematic illustration of the evoked fMRIexperimental design on the EPI-BOLD activation map of FP-S1 regionoverlaid on an anatomical RARE image. (B-C) Schematic drawing of GELINEimaging (B) and an acquired 2D image (100 μm resolution) ofGELINE (C). (D) Two representative 2D line-profiles of GELINE (averageof 40 voxels): good saturation (green arrow) and bad saturation (purplearrow). Error bars represent mean ± SD across thecortical depths (0-2 mm). (E-F) Schematic drawing of SELINE imaging (E)and an acquired 2D image (100 μm resolution) of SELINE (F). (G)two representative 2D line-profiles of SELINE (average of 40 voxels):good saturation (green arrows). Error bars representmean ± SD across the cortical depths (0-2 mm). (H)tSNR comparison between GELINE and SELINE (t-test:^*^p<10^-12^). (I-J) A representative trial of GELINE.(I)*Top*: Demeaned fMRI time series (32 epochs, 10 min40 s) of raw (black) and filtered (red) data (average of 40 voxels,bandpass: 0.01-0.1 Hz) in the FP-S1 region during electrical stimulation(3 Hz, 4 s, 2.5 mA) to left forepaw.*Bottom*: Normalizedspatiotemporal map of the laminar-specific responses along the corticaldepths (0–2 mm, 50 μm resolution). (J)*Top*: Average BOLD time courses and*Bottom*: Average percentage change map across thecortical depths (0–2 mm, 40 lines in total) in the FP-S1. (K-L) Arepresentative trial of SELINE. (K)*Top*: Demeaned fMRItime series (32 epochs, 10 min 40 s) of raw (black) and filtered (red)data (average of 40 voxels, bandpass: 0.01–0.1 Hz) in the FP-S1region during electrical stimulation (3 Hz, 4 s, 2.5 mA) to leftforepaw.*Bottom*: Normalized spatiotemporal map of thelaminar-specific responses along the cortical depths (0–2 mm, 50μm resolution). (L)*Top*: Average BOLD timecourses and*Bottom*: Average percentage change mapacross the cortical depths (0–2 mm, 40 lines in total) in theFP-S1. Pink arrows indicate peak BOLD signals across the corticallayers.

All data sets from rats were acquired using a 14.1T/26 cm (Magnex, Oxford)horizontal bore magnet with an Avance III console (Bruker, Ettlingen) and a 12cm diameter gradient system (100 G/cm, 150 μs rising time). A home-madeRF transceiver surface coil with a 10 mm diameter was used on the rat brain. Forthe functional map of BOLD activation (Fig.1A), a 3D gradient-echo EPI sequence was acquired with the followingparameters: TR/TE 1500/11.5 ms, FOV 1.92 × 1.92 × 1.92cm^3^, matrix size 48 × 48 × 48, spatial resolution0.4 × 0.4 × 0.4 mm^3^, and readout bandwidth 133928 Hz. Ahigh order (e.g., 2^nd^or 3^rd^order) shimming was appliedto reduce the main magnetic field (B0) inhomogeneities at theregion-of-interest. For anatomical reference of the activated BOLD map, a RAREsequence was applied to acquire 48 coronal images with the same geometry as thatof the EPI images. The fMRI design paradigm for each trial comprised 200 dummyscans to reach steady-state, 10 pre-stimulation scans, 3 scans duringstimulation, and 12 post-stimulation scans with a total of 8 epochs.

### 2.3 GELINE acquisition

GELINE datasets (9 trials of 4 rats) were acquired with a 6-mm diameter home-madetransceiver surface coil in anesthetized rats for evoked fMRI. GELINE wasapplied by using two saturation slices to avoid aliasing artifacts in thereduced field-of-view along the phase encoding (i.e., from left to right)direction (Fig. 1Band1C). 2D line profiles were acquired to evaluate saturationRF pulses performance (Fig. 1D). Thedetails of the saturation RF pulse were as follows: pulse shape sech.exc(adiabatic) installed on Bruker PV 5.1, length 1 ms, bandwidth 20250 Hz, FA90°, bandwidth factor of the pulse 20250 Hz·ms, normalized shapeintegral 0.106428, rephasing factor 50 %, and derived power 0.2048 W. LaminarfMRI responses were acquired along the frequency-encoding direction (Fig. 1Iand1J). The following acquisition parameters were used: TR/TE 100/12.5ms, TA 10 min 40 s, FA 50°, slice thickness 1.2 mm, FOV 6.4 × 1.2mm^2^, 1D readout matrix 128 (for the 2D line profiles, FOV 6.4× 12.8 mm^2^, matrix 64 × 128), and readout bandwidth9014 Hz. The fMRI design paradigm for each epoch consisted of 1 spre-stimulation, 4 s stimulation, and 15 s post-stimulation within a total of 20s. A total of 6400 lines (i.e., 10 min 40 s) in each cortex were acquired everysingle trial in evoked fMRI. Evoked BOLD activation was induced by performingelectrical stimulation to the left forepaw (300 µs duration at 2.5 mArepeated at 3 Hz for 4 s). A GELINE 2D image was additionally acquired with andwithout outer volume suppression (Figs. S2 and S3A). For this 2D image, 14T/13 cm (MagnexScientific, horizontal bore) and a 6 cm diameter gradient system (100 G/cm, 150μs rising time) was additionally used. A home-made RF transceiver surfacecoil with a 25 mm diameter was used on the rat brain. The following acquisitionparameters were used: for without outer volume suppression, TR/TE 100/12.5 ms,TA 2 min 33 s, Average 4, FA 30°, slice thickness 1.2 mm, FOV 19.2× 19.2 mm^2^, and matrix 384 × 384, and readout bandwidth17857 Hz; for with outer volume suppression, TR/TE 100/12.5 ms, TA 2 min 33 s,Average 4, FA 30°, slice thickness 1.2 mm, FOV 25.6 × 25.6mm^2^, and matrix 128 × 128, and readout bandwidth 9091Hz.

### 2.4 SELINE acquisition

SELINE datasets (18 trials of 4 rats) were acquired in anesthetized rats forevoked fMRI. SELINE was applied by the 180˚ RF pulse orientedperpendicular to the α˚ excitation RF pulse as moving therefocusing gradient to phase encoding gradient in order to obtain high spatialresolution without reduced FOV aliasing problem along the phase encoding (i.e.,from left to right) direction (Fig. 1Eand1F). 2D line profiles were alsoacquired to evaluate the refocusing RF pulses performance (Fig. 1G). Laminar fMRI responses were acquired along thefrequency-encoding direction (Fig. 1Kand1L). The following acquisitionparameters were used: for 1000 ms SELINE acquisition, TR/TE/FA 1000/20ms/90°, TA 10 min 40 s, slice thickness 1.2 mm, FOV 3.2 × 1.2mm^2^, and 1D readout matrix 64 (for the 2D line profiles, FOV 6.4× 12.8 mm^2^, matrix 64 × 128), and readout bandwidth5000 Hz; for 200 ms SELINE acquisition, TR/TE/FA 200/10 ms/100° or130° or 150°, TA 10 min 40 s, slice thickness 1.2 mm, FOV 6.4× 1.2 mm^2^, 1D readout matrix 64, and readout bandwidth 9014Hz. A SELINE 2D image was also acquired with and without inner volumesuppression (Figs. S2 andS3B) on the 14T MRI (Magnex Scientific) used to acquire the 2D GELINE(Figs. S2 andS3A). The following acquisition parameters were used: for without outervolume suppression, TR/TE 200/10 ms, TA 2 min 33 s, Average 4, FA 150°,slice thickness 1.2 mm, FOV 19.2 × 19.2 mm^2^, and matrix 192× 192, readout bandwidth 26455 Hz; for with outer volume suppression;TR/TE 200/10 ms, TA 2 min 33 s, Average 4, FA 150°, slice thickness 1.2mm, FOV 12.8 × 12.8 mm^2^, and matrix 128 × 128, andreadout bandwidth 10000 Hz. The fMRI experiment set-up was identical to those ofthe GELINE in evoked fMRI.

### 2.5 Data analysis

All signal processing and analyses were implemented in MATLAB software(Mathworks, Natick, MA) and Analysis of Functional NeuroImages software (Cox, 1996) (AFNI, NIH, USA). For evokedfMRI analysis forFigure 1A, thehemodynamic response function (HRF) used was the default of the block functionof the linear program 3dDeconvolve in AFNI. BLOCK (L, 1) computes a convolutionof a square wave of duration L and makes a peak amplitude of block response= 1, with$g\left(t\right)=t^{4}e^{-t}/[4^{4}e^{-4}]$. Each beta weightrepresents the peak height of the corresponding BLOCK curve for that class. TheHRF model was defined as follows:

$$HRF\left(t\right)=\overset{\text{min}\left(t,L\right)}{\underset{0}{\int }}g\left(s\right)ds$$

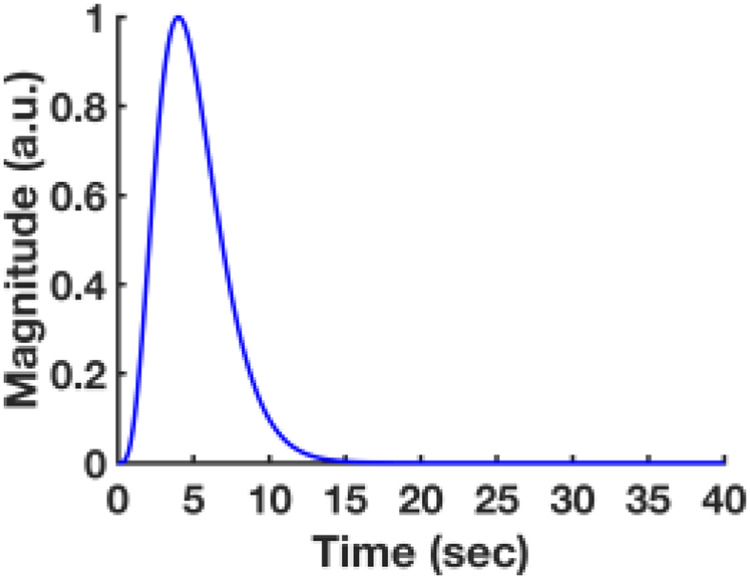


Cortical surfaces were determined based on signal intensities of fMRI lineprofiles as described in the previous work. The detailed processing wasconducted as provided in the previous line-scanning studies (S. Choi, Chen, et al., 2023;S. Choi, Zeng, et al., 2022;X. Yu et al., 2014). For quantitative comparison ofbackground signals between GELINE and SELINE (Fig.1Dand1G), the backgroundsignals were calculated as follows: N_bkg_/S_roi_× 100%, where N_bkg_was the mean of the outside-of-ROI signals andS_roi_was the mean of the ROI signals at the cortical regions. ForFigure 1Iand1K, demeaned fMRI time courses were used as follows: (x -μ), where x was the original fMRI time courses, and μ was the meanof the time courses. The line profile map concatenated with the multiple fMRIsignals was normalized by a maximum intensity (Fig. 1I,1K,3A, and3C; bottom). ForFigure 3F,laminar-specific BOLD signals were normalized by a maximum intensity (i.e., amaximum mean value plus its standard deviation) for both GELINE and SELINE. TheZ-score normalized time courses were calculated as follows: (x -μ)/σ, where x was original fMRI time courses and μ,σ were the mean and the standard deviation of the time courses,respectively (zscore function in MATLAB). Average BOLD time series andpercentage changes were defined as (S-S0)/S0 × 100 %, where S was theBOLD signal and S0 was the baseline. S0 was obtained by averaging thefluctuation signal in the 1-s pre-stimulation window in evoked fMRI that wasrepeated every 20 s with the whole time series (640 s). The BOLD time series ineach ROI were detrended (“polyfit” function in Matlab, order: 3)and bandpass filtered (0.01–0.1 Hz, FIR filter, order: 4096). Thebandpass filtering was performed as a zero-phase filter by “fir1”and “filter” functions in Matlab, compensating a group delay(“grpdelay” and “circshift” functions in Matlab)introduced by the FIR filter. Temporal signal-to-noise ratio (tSNR) values werecalculated across the cortical depths to compare tSNR differences between GELINEand SELINE. Note that σ was calculated as the standard deviation of thewhole time series. We did not use the standard deviation of the time coursesfrom repeated baseline periods because, given our evoked fMRI design paradigm,the baseline period may not be long enough to be considered as resting state (4s stimulation vs. 16 s) (Chen & Pike,2009;Mandeville et al., 1999)and thus could be influenced by the post-stimulus undershoot fluctuations. Thiscalculation for the standard deviation may alter the Z-score normalized timecourses and tSNR values. Student t-test was performed with the tSNR values ofGELINE and SELINE (Fig. 1H). The p-values<0.05 were considered statistically significant.

### 2.6 Steady-state signal simulation

To optimize the α˚ (alpha) excitation flip angle with short TR(i.e., 200 ms) in SELINE, signal intensities were calculated as a function of anexcitation flip angle by solving the Bloch equation (Blenman et al., 2006;Diiokio et al., 1995), by employing the refocusing 180˚ RFpulse. The maximum signal intensity occurred at the optimal angles which wasdefined as follows:

$$S_{xy}\left(\alpha ,\beta \right)=\frac{\sin\left(\alpha \right)\;\cdot \;[1-\cos\left(\beta \right)\;\cdot \;{\text{e}}^{-TR/T1}-\left\{1-\cos\left(\beta \right)\right\}\;\cdot \;{\text{e}}^{-\left(TR-TE/2\right)/T1}]}{1-\cos\left(\alpha \right)\;\cdot \;\cos\left(\beta \right)\;\cdot \;{\text{e}}^{-TR/T1}}\;\cdot \;{\text{e}}^{-TE/T2}$$

whereα,βindicate excitation and refocusing flip angles, respectively, and T1, T2indicate longitudinal and transverse magnetization parameters, respectively. ForGELINE, a steady-state signal was calculated as a function of an excitation flipangle by solving the Bloch equation (Ernst& Anderson, 1966) and defined as follows:

$$S_{xy}\left(\theta \right)=\frac{\sin\left(\theta \right)\;\cdot \;\{1-{\text{e}}^{-TR/T1}\}}{1-\cos\left(\theta \right)\;\cdot \;{\text{e}}^{-TR/T1}}\;\cdot \;{\text{e}}^{-TE/T2^{\text{*}}}$$

whereθindicates an excitation flip angle. T1 and T2 values (i.e., 2211 and 24 ms ofsomatosensory cortex at 16.4T, respectively) were estimated from the previousstudy (Pohmann et al., 2011) because therelaxation values should not be much different from those at 14T.

## 3 Results

### 3.1 Mapping the evoked BOLD fMRI signals with GELINE and SELINE

We developed the SELINE method to map laminar-specific BOLD responses acrosscortical layers at the primary forepaw somatosensory cortex (FP-S1) ofanesthetized rats, which could be compared with the conventional GELINE method(X. Yu et al., 2014). First,unilateral electrical stimulation of the left forepaw of rats showed robust BOLDresponses in the right FP-S1 using EPI-fMRI method (Grandjean et al., 2023) (Fig. 1A). Using the GELINE method, the selected FOV wasdefined by two saturation slices to avoid the aliasing problem along the phaseencoding direction (Fig. 1B). In contrast,the same FOV could be selected by applying a refocusing 180° RF pulseperpendicular to the excitation slice with SELINE (Fig. 1E). To compare ROI selectivity between GELINE and SELINE, 2Din-plane images were acquired by turning on a phase encoding gradient (Fig. 1Cand1F) and 1D profiles were plotted by averaging all readout voxels ofthe 2D image (Fig. 1Dand1G). Full width at half maximum (FWHM) of the1D profiles was estimated: For GELINE, trial #1) and #2) ~1.8 mm, and forSELINE: trial #1) and #2) ~1.5 mm. Background signals were estimated from theareas outside of the FOV (for details, see theMethodsection): For GELINE, trial #1) 10.6 %, #2) 15.8 %, and forSELINE: trial #1) 4.9 %, #2) 5.0 %. The variation in the background signalsuppression of GELINE was potentially caused by imperfect saturation RF pulsesand B0 field inhomogeneity in the outside of the ROI (Fig. S2). This resultindicated the efficiency of the SELINE method to produce sharper 2D sliceprofiles and lower background signals.

To study the laminar fMRI characteristics of GELINE and SELINE across thecortical layers, we calculated tSNR with 1D line-profiles which were acquired byturning off the phase encoding gradient. The tSNR of SELINE was higher thanthose of GELINE (Fig. 1H). The tSNR graphof SELINE showed a gradually decreasing trend across the cortical depth whilethose of GELINE showed a gradually increasing trend. To predict tSNR differenceby solving the Bloch equation (see theMethods), we calculated theoretical tSNR with steady-state signalsgiven T1 (~2200 ms), T2 (~24 ms), and T2* (~20 ms) values and scanparameters such as TRs (1000 ms vs. 100 ms), TEs (20 ms vs. 12.5 ms), flipangles (90° vs. 50°), readout bandwidths (5000 Hz vs. 9014 Hz),and readout FOVs (3.2 mm vs. 6.4 mm), assuming tSNR increased linearly with SNR(Han et al., 2019) and noisecontribution to BOLD signals was identical in both acquisitions. The ratio ofthe simulated tSNR of SELINE vs. GELINE was ~2.2 (~7.0 x 10^-6^/3.2 x10^-6^) while the ratio of the experimental tSNR was ~1.6(~50.8/31.0). In this simulation, we did not consider different effects of noisesources (e.g., thermal noises, physiological noises, and background noises fromoutside of ROI) (Khatamian et al., 2016;Krüger & Glover, 2001;Ragot & Chen, 2019) which wasalso likely to lead to the difference not only between the simulated andexperimental results but also between GELINE and SELINE. It should be noted thatthe transceiver surface coil caused a non-uniform B1 field and possiblycontributed to the tSNR difference between GELINE and SELINE across corticaldepths.

As shown inFigure 1I-L, we demonstrateddynamic BOLD responses across different cortical layers of FP-S1 from arepresentative trial in individual GELINE (Fig.1Iand1J) and SELINE (Fig. 1Kand1L) studies.Figure 1Idemonstrated periodic evoked BOLD signals upon left forepaw electricalstimulation with the T2*-weighted GELINE method, showing the dynamiclaminar-specific BOLD responses as a function of time peaked around thesuperficial layer in the FP-S1 (4 s on/16 s off for each 20 s epoch, total 32epochs). Average BOLD time series and laminar-specific BOLD maps illustratedthat the peak BOLD response is located at L1, highlighting large draining veineffects at the cortical surface (J. Goense etal., 2016;Goense &Logothetis, 2006;Han et al.,2019,^2021^;Polimeni et al., 2010;Zhao et al., 2004,^2006^) (Fig. 1J). In comparisonto GELINE, SELINE also detected robust FP-S1 BOLD signals across differentcortical layers (Fig. 1K), but showed thepeak BOLD signal located at L4, presenting improved spatial specificity todeeper cortical layers (J. Goense et al.,2016;Goense & Logothetis,2006;Han et al., 2019,^2021^;Harelet al., 2006;Zhao et al.,2004,^2006^) and similar BOLDsignal change with a longer TR (1000 ms) (Fig.1L).

### 3.2 Comparison of the laminar-specific peak BOLD responses in GELINE and SELINE

**Fig. 2.**
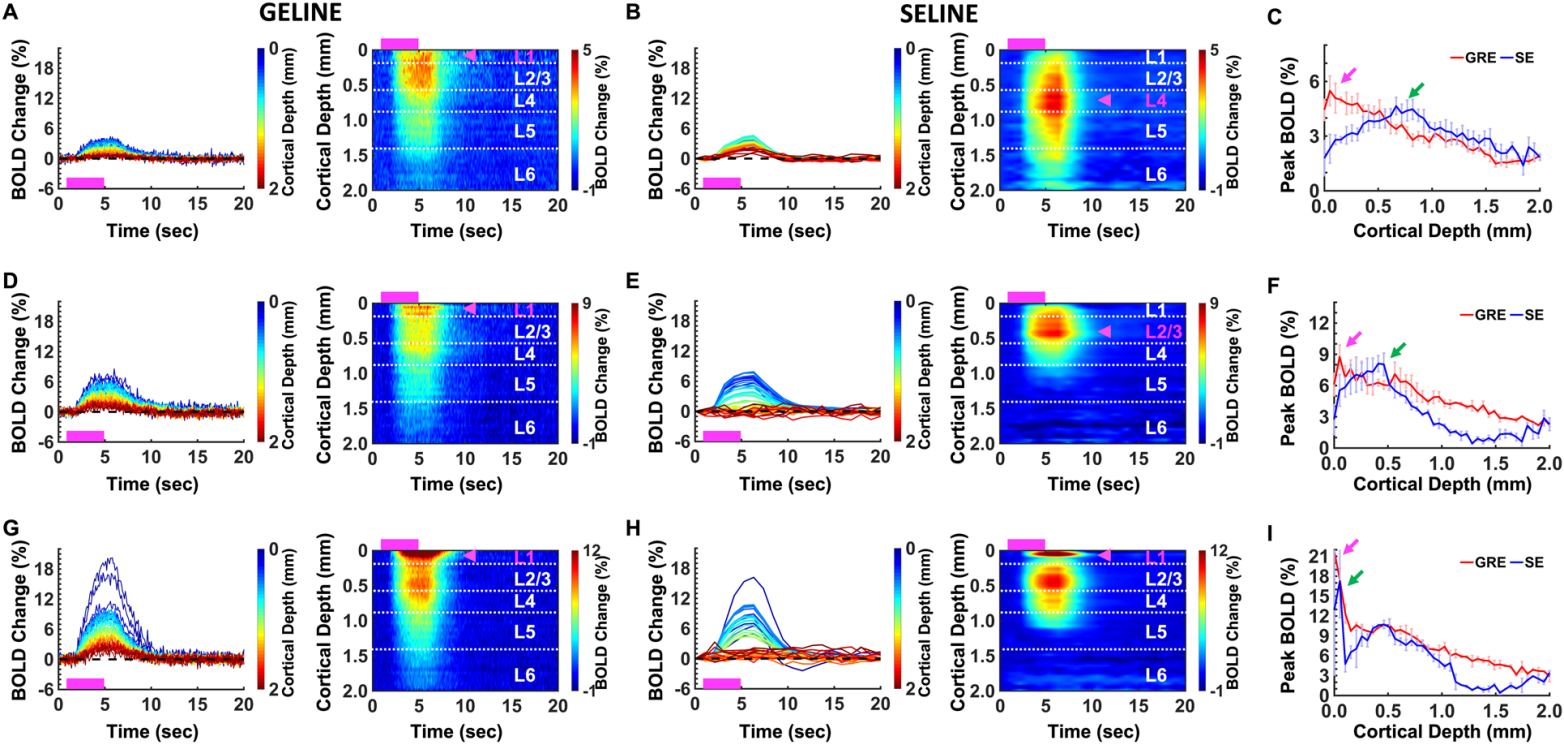
Evoked fMRI time series and percentage change maps of GELINE and SELINEin rat brains (14 trials of 3 rats). (A-C). Rat #1 (3 trials of each).(D-F). Rat #2 (2 trials of each). (G-I). Rat #3 (2 trials of each). (A,D, G)*Left*: Average BOLD time courses and*Right*: Average percentage change map of GELINEacross the cortical depths (0–2 mm, 40 lines in total) in FP-S1region. (B, E, H)*Left*: Average BOLD time courses and*Right*: Average percentage change map of SELINEacross the cortical depths (0–2 mm, 40 lines in total) in FP-S1region. Pink boxes indicate stimulation duration and pink arrowsindicate peak BOLD signals across the cortical layers. (C, F, I)Comparison of peak BOLD signals between GELINE (pink arrows) and SELINE(green arrows). Error bars represent mean ± SD ofpeak BOLD signals.

We further investigated the reproducibility of laminar-specific peak BOLDresponses, as well as the variability of laminar-specific BOLD responsepatterns, between the two methods (14 trials from 3 animals). The GELINE methoddetected peak BOLD signals primarily located at L1, but the peak BOLD signaldetected by the SELINE method was much deeper (Fig. 2). In animal #3, the strong BOLD signal detected in thesuperficial voxel indicates a large draining vein dominating the voxel BOLDsignal (Fig. 2G,2H, and2I). A similarBOLD response was also detected by the SELINE method, which is most likely tostem from non-negligible intravascular effects of the large draining vein in thesuperficial voxels with only 50 μm thickness or increased motionalnarrowing effects due to the fast diffusion rate of spins from cerebrospinalfluid (CSF) (Pfaffenrot et al., 2021;Uludaǧ et al., 2009).

Interestingly, the layer-specific BOLD signal varied largely across animals inboth GELINE and SELINE maps. Besides the primary BOLD peak in L1 of GELINE, asecond peak appeared in L4 in some animal (Fig.2Dand2F). And for the SELINEmethod, the primary peak also varied at L4 and L2/3 (Fig. 2B,2C,2E, and2F), which presented highly different laminar patterns from GELINEeven when acquired from the same animal with interleaved trials duringexperiments (Fig. 2Aand2D). These results have suggested that theprofile of laminar-specific BOLD signals can vary largely across animals, whichmay present varied dynamic patterns of BOLD responses due to the alteredneurovascular coupling across different cortical layers.

### 3.3 Mapping the laminar BOLD responses with a 200 ms SELINE method

**Fig. 3.**
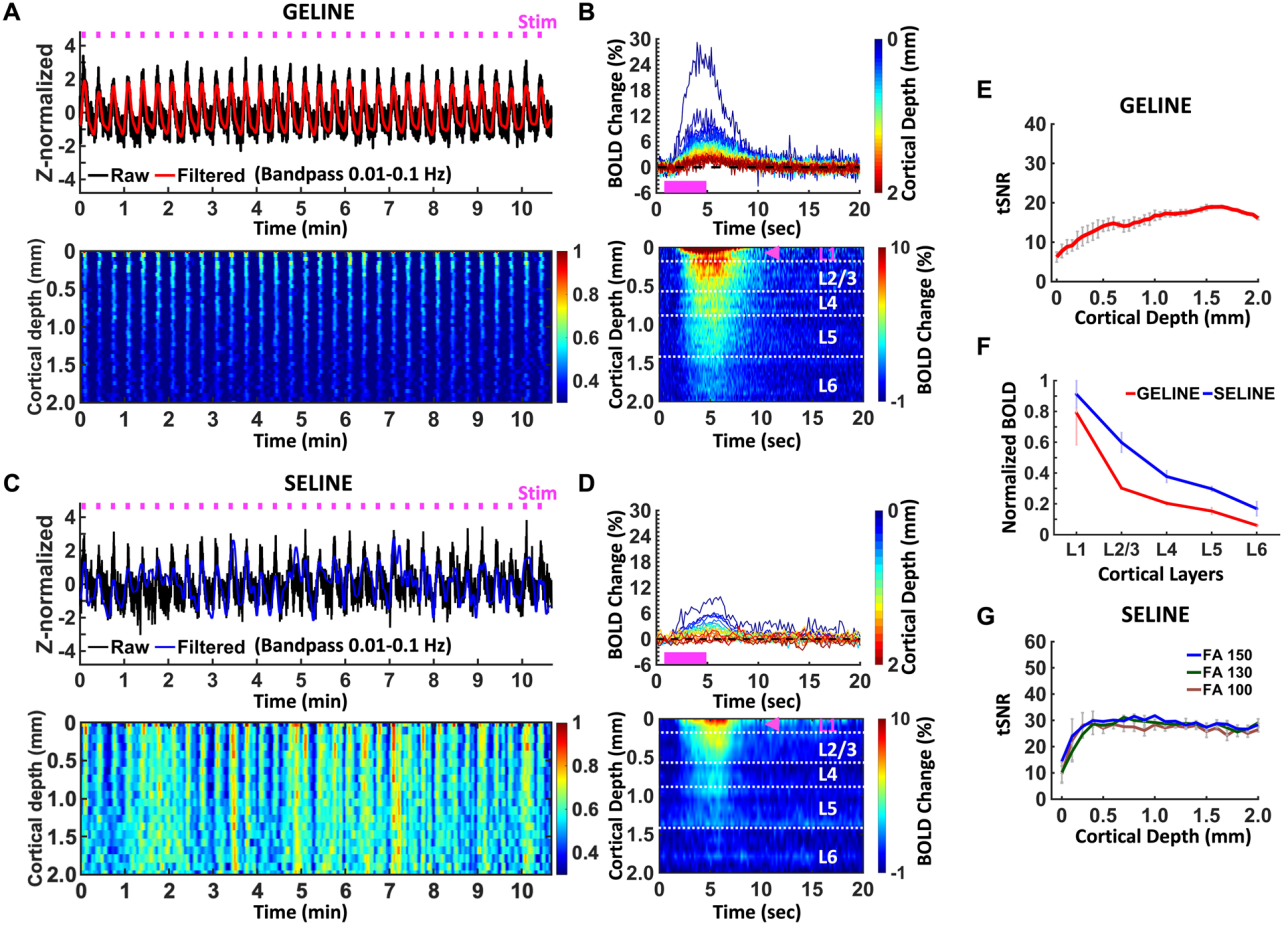
Evoked fMRI responses with GELINE (TR 100 ms) versus SELINE (TR 200 ms).(A-B) GELINE (2 trials) (A)*Top*: Z-score normalizedfMRI time series (average of 40 voxels) of FP-S1.*Bottom*: Normalized spatiotemporal map of thelaminar-specific responses along the cortical depths (0–2 mm, 50μm resolution). (B)*Top*: Average BOLD timecourses and*Bottom*: Average percentage change mapacross the cortical depths (0–2 mm, 40 lines in total) in theFP-S1. (C-D) SELINE (3 trials, FA 150°). (C)*Top*: Z-score normalized fMRI time series (average of 20voxels) of FP-S1.*Bottom*: Normalized spatiotemporal mapof the laminar-specific responses along the cortical depths (0–2mm, 100 μm resolution). (D)*Top*: Average BOLDtime courses and*Bottom*: Average percentage change mapacross the cortical depths (0–2 mm, 20 lines in total) in theFP-S1. (E) tSNR of GELINE (mean 15.3, 2 trials) across the corticaldepths (0–2 mm). (F) Comparison of normalized BOLD signalsbetween GELINE and SELINE across cortical layers. (G) tSNR comparison ofSELINE with three excitation flip angles across the cortical depths(0–2 mm): FA 100° (mean 26.0, 5 trials), FA 130°(mean 26.9, 3 trials), and FA 150° (mean 28.1, 3 trials) afterB1- inhomogeneity correction (Fig. S1). Pink boxes indicate stimulationduration and pink arrows indicate peak BOLD signals across the corticallayers. Error bars represent mean ± SD.

We performed BOLD fMRI experiments with a 200 ms TR by applying optimized flipangles based on the Bloch equation (Blenman etal., 2006;Diiokio et al.,1995) (see theMethodsection).For comparison, we also performed the GELINE method in the same anesthetizedrat. As shown inFigure 3A-D, wedemonstrated the evoked BOLD responses across the cortical layers upon theperiodic electrical stimulation with the GELINE (Fig. 3Aand3B) and SELINE(Fig. 3Cand3D) methods, showing the average BOLD time series andpercentage changes peaked at L1 in both GELINE and SELINE. To characterize thelaminar-specific BOLD responses, the normalized BOLD signals were plotted acrossthe cortical layers. As shown inFigure 3F,the GELINE method had a steep signal drop from L1 to L2/3, while the SELINEmethod had a gradual signal drop across the cortical depth. Although thelaminar-specific BOLD responses of the 200 ms SELINE maintained large vesselssensitivity in the superficial layers (Fig.3D), the peak BOLD signal of the SELINE were much lower than that ofthe GELINE: ~10 % vs. ~30 % and the slopes of the normalized BOLD signal plotillustrated that the SELINE method had less bias to large draining veins thanthe GELINE method at the superficial layers (slope at L1 and L2/3: SELINE; -0.31vs. GELINE; -0.49). Taken together, these results indicate that the hightemporal SELINE method reduces the large vessel contribution to the BOLDresponses by minimizing magnetic susceptibility effects at the superficiallayers (i.e., L1 and L2/3).

To select an optimized flip angle, the tSNR of different flip angles was plotted(Fig. 3G). Even though the optimal flipangle for TR 200 ms was ~150° and had the highest tSNR, the difference ofthe tSNR change was relatively small in multiple trials with the different flipangles regardless of B1^-^inhomogeneity correction (Fig. 3GandFig. S1) (Delgado et al., 2020). This result waspossibly caused by the long T1 effect (~2200 ms) of the cortex in the SELINEacquisition with a short TR (200 ms) (Pohmann etal., 2011). Same as the theoretical predictions based on the Blochequation (Blenman et al., 2006;Diiokio et al., 1995),${\text{e}}^{-TR/T1}$was almostclose to 1 and thus, the maximum intensity at the optimal flip angle did notchange a lot. Average tSNR values for GELINE and SELINE were 15.3 and 27.0 whilethe tSNR efficiency of those was 48.4 and 60.4, respectively. It is noteworthythat the average tSNR of SELINE was higher at the superficial and middle layersthan that of GELINE (Fig. 3Eand3G) due to a larger flip angle(100-150° vs. 50°) and longer TR (200 ms vs. 100 ms). In summary,these results not only demonstrated less magnetic susceptibility effects at thesuperficial layer, but also highlighted laminar specificity enhancement inSELINE with high temporal resolution.

## 4 Discussion

In this study, we applied the SELINE method to investigate laminar-specific evokedBOLD responses across cortical layers with high spatial and temporal resolution. TheSELINE method has sharper and better ROI-selectivity than the GELINE method,employing the refocusing 180° RF pulse perpendicular to the excitation plane.It should be noted that a part of tissue on the upper side of the 2D SELINE image(Fig. 1F) remained outside the ROI and wasseverely distorted due to nonlinear gradient around the air-tissue boundary (i.e.,skull) and chemical shift of fat signals (Sakurai etal., 1992) at an ultra-high magnetic field (14.1T). Since our analysesfocused on cortical layers (0–2 mm) and the varying displacement resulted ingeometric distortion occurred outside of the cortex, the influence of the imperfectsuppression was negligible. As ascertained in the 2D GELINE image (Fig. 1C), a major signal source of the pile-updisplacement artifact was fat tissue. This issue thus can be alleviated by applyingfat suppression pulses and high readout bandwidth (Raimondo, Heij, et al., 2023;Sakurai etal., 1992). Our results show that the peak signal of SELINE is spreadacross the cortical layers while that of GELINE is at the superficial layer (Han et al., 2019;Krüger & Glover, 2001). By pushing thetemporal resolution of SELINE to 200 ms, we also demonstrate the feasibility to maplaminar-specific BOLD responses with less large draining vein effects (Boxerman et al., 1995;J. Goense et al., 2016;Goense & Logothetis, 2006;Han etal., 2019,^2021^;Weisskoff et al., 1994;Yacoub et al., 2003,^2005^;Zhao et al., 2004,^2006^), in comparison to the GELINE method.

Significant effort with high-field fMRI has been made to explore laminar fMRIresponses corresponding to distinct information flows (e.g., top-down/bottom-up orfeedforward/feedback) at high spatial and temporal scales in both animals andhumans. Among these efforts detecting BOLD, cerebral blood volume (CBV), andcerebral blood flow (CBF) signals with both SE and GRE methods, corticaldepth-dependent fMRI has identified hemodynamic regulation, blood volumedistribution, circuit-specific laminar responses, and hierarchical informationstreams across cortical layers in animal (Albers etal., 2018;S. Choi, Chen, et al.,2023;S. Choi, Zeng, et al., 2022;J. B. Goense & Logothetis, 2006;Jung et al., 2021;Lu et al., 2004;Shen& Duong, 2016;Silva &Koretsky, 2002;X. Yu et al.,2014;Zhao et al., 2004,^2006^) and human brains (Finn et al., 2019;Huberet al., 2017;Kashyap et al.,2018;Sharoh et al., 2019;Y. Yu et al., 2019). In particular,high-resolution CBV-fMRI, based on the VASO mapping scheme, has been used to measurelayer-specific directional functional connectivity across human motor cortex andsomatosensory and premotor regions (Huber et al.,2017). It should be noted that the cortical thickness of human brains isin the range of 1–4 mm, which is highly comparable to that of rodent brainsin the range of 1–2 mm (Fischl & Dale,2000). Given the limited spatial resolution of the high fieldlaminar-fMRI method (~600-700 um), the thoroughly counted voxels across differentcortical depth regions are in the single digit number. This could be much betterimproved by the developed line-scanning fMRI method, as well as with ultra-fastsampling rates that enable the detection of fast hemodynamic responses acrosscortical layers without compounding artifacts (Caballero-Gaudes & Reynolds, 2017).

Recently, the GRE-based line-scanning BOLD mapping scheme has been implemented toinvestigate BOLD signals across cortical layers in human fMRI studies (Morgan et al., 2020;Raimondo et al., 2021;Raimondo, Priovoulos, et al., 2023). Nevertheless, since SAR isproportional to the square of the magnetic field (B0) and the duty cycle of thesequence, high temporal resolution in ultra-high-field fMRI studies can beconstrained by SAR limits especially representing a safety-related limit inhigh-field human MRI system (e.g., 7T and 9.4T). Compared to the SELINE method, therequired saturation RF pulses of the GELINE method theoretically result in higherSAR and total RF power limits with short TRs, inducing more complicated aliasingproblems. For the SELINE method, the beam-like line-scan projection has beenpreviously applied for probing myeloarchitecture across cortical layers in theprimary somatosensory cortex (S1) and the primary motor cortex (M1) of the humanbrain (Balasubramanian et al., 2021) andmapping irreversible and reversible transverse relaxation rates (i.e., R2 andR2´) in primary visual cortex (V1), S1, and M1 of human brains (Balasubramanian et al., 2022). The sharperline-profile has been also demonstrated in human fMRI studies by employing theSELINE method at a cost of compromising tSNR and BOLD sensitivity (Raimondo, Heij, et al., 2023). Although pre- andpost-data processing steps (i.e., NORDIC (Vizioli etal., 2021), SNR-optimized coil combination (S. Choi et al., 2016;Raimondo et al., 2021;Roemer et al.,1990), and independent component analysis (Mckeown et al., 2003)) were applied to enhance tSNR andfunctional sensitivity, no task-driven activation was observed in this initial humanfMRI study (Raimondo, Heij, et al., 2023).This suggests that prospective motion correction (Bause et al., 2020), averaging of more runs (Huettel & Mccarthy, 2001), and localized surfacecoils (Kashyap et al., 2018), which were notincorporated into this study, are required for the future work. In contrast, theprevious large-tip-angle spin-echo line-scanning fMRI study in anesthetized ratselucidated that early onset of laminar-specific BOLD responses occurred at themiddle layers for comparison of diffusion fMRI onset while potential aliasing issuesof saturation RF pulses remained (Nunes et al.,2021). We thus applied this SELINE method to better characterizelayer-specific fMRI features across cortical depths at FP-S1 of rodent brainswithout the need for additional saturation RF pulses. The SELINE method employed thespin-echo scheme to reduce the large draining vein effect, which could be furtherdistinguished from the deeper cortical layer responses given the high spatialresolution (Fig. 1I–L).

As reported in previous studies (Duong et al.,2003;J. Goense et al., 2016;J. B. Goense & Logothetis, 2006;Yacoub et al., 2003,^2005^;Zhao et al.,2004,^2006^), GELINE is moresensitive to large veins at the pial surface but has poor specificity acrossdifferent cortical depths, whereas SELINE is less vulnerable to superficial largedraining veins but has good sensitivity to micro-vessel across cortical layers.However, the largely varied laminar patterns of the BOLD responses were observed inboth methods (Fig. 2). This may suggest thatthe varied patterns of laminar-specific BOLD signals pertain to microvascular biasesand baseline blood volume distribution across cortical layers (Hartung et al., 2022a,2022b). In addition, TE is crucial for optimizing the SELINE BOLDresponses because the relative micro- and macro-vascular contributions to BOLDsignals can be changed by altering the TE (Boxermanet al., 1995;Uludaǧ et al.,2009). Here, we chose a TE of 20 ms (for a TR of 1000 ms) which wasanalogous to the tissue T2 value (~20 ms) to minimize macrovascular contribution tothe BOLD responses (Pohmann et al., 2011).For high temporal SELINE acquisition (TR 200 ms), a TE of 10 ms was chosen tooptimize macrovessel suppression and tSNR preservation. Also, the TEs wererelatively long compared to the venous blood T2 value at 14T (<10 ms) (Lin et al., 2012), resulting in smallintravascular contribution to the SELINE BOLD responses. Note that, nevertheless,intravascular SE-based BOLD signals do not completely disappear even at high-fieldMRI when the partial volume contribution of vessels to the given voxel is notnegligible (Uludaǧ et al., 2009). Insome cases of SELINE, strong BOLD responses at the superficial layer existed (Fig. 2Hand3D). As accepting that both intra- and extra-vascular signals contributeto the total BOLD responses, it is most likely to originate from the followingfactors: 1) The extravascular effect in combination with non-negligible cerebralblood volume effect from large vessels can be dominant in the GRE-based acquisitionscheme. Depending on the surface draining vein and diving artery localization, thesuperficial voxels can be heavily influenced by the extravascular effects, as wellas active or passive vessel dilation effect. The combined signal changes coulddramatically influence the signal changes in the GRE scheme. 2) The non-negligibleintravascular effects can remain in the SE-based acquisition scheme for superficialvoxels with large partial volume contribution from vessels (Boxerman et al., 1995). In these superficial voxels, thevessel volume contribution is far higher than 2-4% as reported for conventional fMRIstudies (Ji et al., 2021;Kim & Ogawa, 2012;Weber et al., 2008), and the direct intravascular effects caused by oxy-and deoxy-hemoglobin ratio changes could contribute to the BOLD signal from thesevoxels. In parallel, the SE scheme would reduce the extravascular effect, whichfurther makes the intravascular effect from vessels less negligible. 3) Variedinflow effects (Axel, 1984) can influencelaminar fMRI signals given the vascular distribution. The blood flow would alter thespin-echo effect given the TE selected for 200 ms and 1000 ms TRs. The spins in theflowing blood would experience less rephasing effect based on the SE scheme and showweaker signals. Nevertheless, if spins excited by a 90º (or αº)RF pulse, originally outside of the line profile, flow into the 180º RFexcitation slice, it would contribute to the line-profile signals erroneously andimpact the laminar BOLD responses. For GRE-acquisition scheme, if flowing freshblood enters into the image plane, new spins would experience an excited RF pulse.With increasing blood flow velocity, the fresh blood signal increases since thetotal number of experienced RF pulses decrease. 4) CSF partial volume effect (Pfaffenrot et al., 2021;Uludaǧ et al., 2009) might occur at superficialvoxels due to slow CSF flow at a TR of 1000 ms. This is similar to the infloweffects discussed in the previous section. Meanwhile, for the superficial voxelswhen CSF took a non-negligible partial volume contribution similar to blood vessels,it could also contribute to the altered laminar BOLD responses. Since the spatialresolution of the fast SELINE method was lower than that of the GELINE method, thelayer boundaries of the laminar response patterns might be vague and blurred in thefast SELINE results (Fig. 3). Whereas thevaried peak profiles of BOLD responses exist across different cortical layerspresumably due to the confounding factors, these results illustrate the feasibilityof the line-scanning method to detect distinct laminar BOLD responses. It provides ahigh-resolution mapping scheme when investigating altered neurovascular couplingevents and functional connectivity across cortical layers.

The main limitation of SELINE is the slow sampling rate. The sampling rate (TR) isdetermined by two times of the TE. TE is generally limited by the duration of theexcitation and refocusing RFs, the duration of the slice rephasing gradient, and theduration of the frequency encoding gradient (i.e., readout matrix size andbandwidth). We attempted to shorten the TR by adjusting the excitation flip angle(α). Based on the Bloch equation (Blenman etal., 2006;Diiokio et al., 1995),we have estimated the appropriate angles with a short TR (i.e., 200 ms). Our resultsshow the feasibility of the fast SELINE method which has a good sampling capacitycapturing dynamic BOLD signals from superficial to deeper layers. For future work,the fast SELINE method should be optimized in terms of spoiling and phase cyclingschemes to enhance tSNR. Furthermore, simultaneous GRE- and SE-type fMRIacquisitions can be applied to better characterize laminar-specific fMRI patternsand minimize time dependency of dynamic fMRI responses by employing GRASE (Oshio & Feinberg, 1991)-basedline-scanning in rodents as already suggested for the human fMRI mapping (S. Choi, Yu, et al., 2022). For laminar humanfMRI studies, a fat suppression RF (e.g., SPIR, SPAIR, STIR, etc.) should be appliedto avoid fat aliasing artifacts in cortical areas at relatively low magnetic fields(e.g., 7T) (Raimondo et al., 2021;Raimondo, Heij, et al., 2023). Imperfect fatsuppression can also be alleviated by adjusting acquisition parameters (e.g.,readout matrix size and bandwidth).

## Supplementary Material

Supplementary Material

## Data Availability

All other data generated during this study are available from the correspondingauthor upon reasonable request. The related image processing codes are availablefrom the corresponding author upon reasonable request.

## References

[b1] Albers , F. , Schmid , F. , Wachsmuth , L. , & Faber , C. ( 2018 ). Line scanning fMRI reveals earlier onset of optogenetically evoked BOLD response in rat somatosensory cortex as compared to sensory stimulation . NeuroImage , 164 , 144 – 154 . 10.1016/j.neuroimage.2016.12.059 28012967

[b2] Axel , L. ( 1984 ). Review blood flow effects in magnetic resonance imaging . American Journal of Roentgenology , 143 ( 6 ), 1157 – 1166 . 10.2214/ajr.143.6.1157 6333785

[b3] Balasubramanian , M. , Mulkern , R. V. , Neil , J. J. , Maier , S. E. , & Polimeni , J. R. ( 2021 ). Probing in vivo cortical myeloarchitecture in humans via line-scan diffusion acquisitions at 7 T with 250-500 micron radial resolution . Magnetic Resonance in Medicine , 85 ( 1 ), 390 – 403 . 10.1002/mrm.28419 32738088 PMC7951328

[b4] Balasubramanian , M. , Mulkern , R. V. , & Polimeni , J. R. ( 2022 ). In vivo irreversible and reversible transverse relaxation rates in human cerebral cortex via line scans at 7 T with 250 micron resolution perpendicular to the cortical surface . Magnetic Resonance Imaging , 90 , 44 – 52 . 10.1016/j.mri.2022.04.001 35398027 PMC9930184

[b5] Bause , J. , Polimeni , J. R. , Stelzer , J. , In , M. H. , Ehses , P. , Kraemer-Fernandez , P. , Aghaeifar , A. , Lacosse , E. , Pohmann , R. , & Scheffler , K. ( 2020 ). Impact of prospective motion correction, distortion correction methods and large vein bias on the spatial accuracy of cortical laminar fMRI at 9.4 Tesla . NeuroImage , 208 , 116434 . 10.1016/j.neuroimage.2019.116434 31812715

[b6] Blenman , M. R.-A. , Port , D. J. , & Felmlee , P. J. ( 2006 ). In vivo large flip angle 31P MRS in the human brain at 3 T . Proceedings of the International Society for Magnetic Resonance in Medicine , 14 , 3088 . https://cds.ismrm.org/protected/06MProceedings/PDFfiles/03088.pdf

[b7] Boxerman , J. L. , Hamberg , L. M. , Rosen , B. R. , & Weisskoff , R. M. ( 1995 ). MR contrast due to intravascular magnetic susceptibility perturbations . Magnetic Resonance in Medicine , 34 ( 4 ), 555 – 566 . 10.1002/mrm.1910340412 8524024

[b8] Budde , J. , Shajan , G. , Zaitsev , M. , Scheffler , K. , & Pohmann , R. ( 2014 ). Functional MRI in human subjects with gradient-echo and spin-echo EPI at 9.4 T . Magnetic Resonance in Medicine , 71 ( 1 ), 209 – 218 . 10.1002/mrm.24656 23447097

[b9] Caballero-Gaudes , C. , & Reynolds , R. C. ( 2017 ). Methods for cleaning the BOLD fMRI signal . NeuroImage , 154 , 128 – 149 . 10.1016/j.neuroimage.2016.12.018 27956209 PMC5466511

[b10] Chen , J. J. , & Pike , G. B. ( 2009 ). Origins of the BOLD post-stimulus undershoot . NeuroImage , 46 ( 3 ), 559 – 568 . 10.1016/j.neuroimage.2009.03.015 19303450

[b11] Choi , S. , Chen , Y. , Zeng , H. , Biswal , B. , & Yu , X. ( 2023 ). Identifying the distinct spectral dynamics of laminar-specific interhemispheric connectivity with bilateral line-scanning fMRI . Journal of Cerebral Blood Flow and Metabolism , 43 ( 7 ), 1115 – 1129 . 10.1177/0271678X231158434 36803280 PMC10291453

[b12] Choi , S. , Park , J.-S. , Kim , H. , & Park , J. ( 2016 ). Image denoising for metal MRI exploiting sparsity and low rank priors . Investigative Magnetic Resonance Imaging , 20 ( 4 ), 215 . 10.13104/imri.2016.20.4.215

[b13] Choi , S. , Xie , Z. , Liu , X. , Zhang , B. , Hike , D. , Liu , A. , & Yu , X. ( 2023 ). Detecting high temporal laminar-specific responses with line-scanning fMRI in awake mice . Proceedings of the International Society for Magnetic Resonance in Medicine . https://submissions.mirasmart.com/ISMRM2023/Itinerary/PresentationDetail.aspx?evdid=455

[b14] Choi , S. , Yu , X. , Scheffler , K. , & Herz , K. ( 2022 ). Simultaneous acquisition of GRE- and SE-type resting-state fMRI signals with GRASE-based line-scanning in the human brain . Proceedings of the International Society for Magnetic Resonance in Medicine , 30 ( 1105 ). https://cds.ismrm.org/protected/22MProceedings/PDFfiles/1105.html

[b15] Choi , S. , Zeng , H. , Chen , Y. , Sobczak , F. , Qian , C. , & Yu , X. ( 2022 ). Laminar-specific functional connectivity mapping with multi-slice line-scanning fMRI . Cerebral Cortex , 32 ( 20 ), 4492 – 4501 . 10.1093/cercor/bhab497 35107125 PMC9574235

[b16] Choi , S. , Zeng , H. , Pohmann , R. , Scheffler , K. , & Yu , X. ( 2018 ). Novel alpha-180 SE based LINE-scanning method (SELINE) for laminar-specific fMRI . Proceedings of the International Society for Magnetic Resonance in Medicine , 27 , 1166 . https://cds.ismrm.org/protected/19MProceedings/PDFfiles/1166.html

[b17] Choi , S.-H. , Im , G. H. , Choi , S. , Yu , X. , Bandettini , P. A. , Menon , R. S. , & Kim , S.-G. ( 2023 ). No replication of direct neuronal activity-related (DIANA) fMRI in anesthetized mice . bioRxiv . 10.1101/2023.05.26.542419 PMC10971415 38536912

[b18] Cox , R. W. ( 1996 ). AFNI: Software for analysis and visualization of functional magnetic resonance neuroimages . Computers and Biomedical Research , 29 ( 3 ), 162 – 173 . 10.1006/cbmr.1996.0014 8812068

[b19] Delgado , P. R. , Kuehne , A. , Periquito , J. S. , Millward , J. M. , Pohlmann , A. , Waiczies , S. , & Niendorf , T. ( 2020 ). B1 inhomogeneity correction of RARE MRI with transceive surface radiofrequency probes . Magnetic Resonance in Medicine , 84 ( 5 ), 2684 – 2701 . 10.1002/mrm.28307 32447779

[b20] Diiokio , G. , Brown , J. J. , Borrello , J. A. , Perman , W. H. , & Shu , H. H. ( 1995 ). Large angle spin-echo imaging . Magnetic Resonance Imaging , 13 ( 1 ), 39 – 44 . 10.1016/0730-725x(94)00082-e 7898278

[b21] Duong , T. Q. , Yacoub , E. , Adriany , G. , Hu , X. , Uǧurbil , K. , & Kim , S. G. ( 2003 ). Microvascular BOLD contribution at 4 and 7 T in the human brain: Gradient-echo and spin-echo fMRI with suppression of blood effects . Magnetic Resonance in Medicine , 49 ( 6 ), 1019 – 1027 . 10.1002/mrm.10472 12768579

[b22] Ernst , R. R. , & Anderson , W. A. ( 1966 ). Application of Fourier transform spectroscopy to magnetic resonance . Review of Scientific Instruments , 37 ( 1 ), 93 – 102 . 10.1063/1.1719961

[b23] Finn , E. S. , Huber , L. , Jangraw , D. C. , Molfese , P. J. , & Bandettini , P. A. ( 2019 ). Layer-dependent activity in human prefrontal cortex during working memory . Nature Neuroscience , 22 ( 10 ), 1687 – 1695 . 10.1038/s41593-019-0487-z 31551596 PMC6764601

[b24] Fischl , B. , & Dale , A. M. ( 2000 ). Measuring the thickness of the human cerebral cortex from magnetic resonance images . Proceedings of the National Academy of Sciences of the United States of America , 97 ( 20 ), 11050 – 11055 . 10.1073/pnas.200033797 10984517 PMC27146

[b25] Goense , J. B. , & Logothetis , N. K. ( 2006 ). Laminar specificity in monkey V1 using high-resolution SE-fMRI . Magnetic Resonance Imaging , 24 ( 4 ), 381 – 392 . 10.1016/j.mri.2005.12.032 16677944

[b26] Goense , J. , Bohraus , Y. , & Logothetis , N. K. ( 2016 ). fMRI at high spatial resolution implications for BOLD-models . Frontiers in Computational Neuroscience , 10 , 66 . 10.3389/fncom.2016.00066 27445782 PMC4923185

[b27] Grandjean , J. , Desrosiers-Gregoire , G. , Anckaerts , C. , Angeles-Valdez , D. , Ayad , F. , Barrière , D. A. , Blockx , I. , Bortel , A. , Broadwater , M. , & Cardoso , B. M. ( 2023 ). A consensus protocol for functional connectivity analysis in the rat brain . Nature Neuroscience . 10.1038/s41593-023-01286-8 PMC10493189 36973511

[b28] Han , S. H. , Eun , S. , Cho , H. J. , Uludaǧ , K. , & Kim , S. G. ( 2021 ). Improvement of sensitivity and specificity for laminar BOLD fMRI with double spin-echo EPI in humans at 7 T . NeuroImage , 241 , 118435 . 10.1016/j.neuroimage.2021.118435 34324976

[b29] Han , S. H. , Son , J. P. , Cho , H. J. , Park , J. Y. , & Kim , S. G. ( 2019 ). Gradient-echo and spin-echo blood oxygenation level–dependent functional MRI at ultrahigh fields of 9.4 and 15.2 Tesla . Magnetic Resonance in Medicine , 81 ( 2 ), 1237 – 1246 . 10.1002/mrm.27457 30183108 PMC6585650

[b30] Harel , N. , Lin , J. , Moeller , S. , Ugurbil , K. , & Yacoub , E. ( 2006 ). Combined imaging-histological study of cortical laminar specificity of fMRI signals . NeuroImage , 29 ( 3 ), 879 – 887 . 10.1016/j.neuroimage.2005.08.016 16194614

[b31] Hartung , G. , Pfannmoeller , J. , Berman , J. L. A. , & Polimeni , R. J. ( 2022a ). Biophysical simulations of BOLD fMRI responses using human vascular anatomical network models . BRAIN Initiative Conference . https://brainmeeting2022.ipostersessions.com/Default.aspx?s=06-E6-A9-B1-C6-1B-E5-16-05-97-6B-2B-DA-A6-84-DC

[b32] Hartung , G. , Pfannmoeller , J. , Berman , J. L. A. , & Polimeni , R. J. ( 2022b ). Simulated fMRI responses using human vascular anatomical network models with varying architecture and dynamics . Proceedings of the International Society for Magnetic Resonance in Medicine , 30 ( 0682 ). https://cds.ismrm.org/protected/22MProceedings/PDFfiles/0682.html

[b33] Hodono , S. , Rideaux , R. , van Kerkoerle , T. , & Cloos , M. A. ( 2023 ). Initial experiences with direct imaging of neuronal activity (DIANA) in humans . Imaging Neuroscience , 1 , 1 – 11 . 10.1162/imag_a_00013 PMC12007528 40799711

[b34] Huber , L. , Handwerker , D. A. , Jangraw , D. C. , Chen , G. , Hall , A. , Stuber , C. , Gonzalez-Castillo , J. , Ivanov , D. , Marrett , S. , Guidi , M. , Goense , J. , Poser , B. A. , & Bandettini , P. A. ( 2017 ). High-resolution CBV-fMRI allows mapping of laminar activity and connectivity of cortical input and output in human M1 . Neuron , 96 ( 6 ), 1253 – 1263 . 10.1016/j.neuron.2017.11.005 29224727 PMC5739950

[b35] Huettel , S. A. , & Mccarthy , G. ( 2001 ). The effects of single-trial averaging upon the spatial extent of fMRI activation . Neuroreport , 12 ( 11 ), 2411 – 2416 . 10.1097/00001756-200108080-00025 11496120

[b36] Ji , X. , Ferreira , T. , Friedman , B. , Liu , R. , Liechty , H. , Bas , E. , Chandrashekar , J. , & Kleinfeld , D. ( 2021 ). Brain microvasculature has a common topology with local differences in geometry that match metabolic load . Neuron , 109 ( 7 ), 1168.e13 – 1187.e13 . 10.1016/j.neuron.2021.02.006 33657412 PMC8525211

[b37] Jung , W. B. , Im , G. H. , Jiang , H. , & Kim , S. G. ( 2021 ). Early fMRI responses to somatosensory and optogenetic stimulation reflect neural information flow . Proceedings of the National Academy of Sciences of the United States of America , 118 ( 11 ), e2023265118 . 10.1073/pnas.2023265118 33836602 PMC7980397

[b38] Kashyap , S. , Ivanov , D. , Havlicek , M. , Sengupta , S. , Poser , B. A. , & Uludağ , K. ( 2018 ). Resolving laminar activation in human V1 using ultra-high spatial resolution fMRI at 7T . Scientific Reports , 8 ( 1 ), 17063 . 10.1038/s41598-018-35333-3 30459391 PMC6244001

[b39] Khatamian , Y. B. , Golestani , A. M. , Ragot , D. M. , & Chen , J. J. ( 2016 ). Spin-echo resting-state functional connectivity in high-susceptibility regions: Accuracy, reliability, and the impact of physiological noise . Brain Connectivity , 6 ( 4 ), 283 – 297 . 10.1089/brain.2015.0365 26842962 PMC5167563

[b40] Kim , S. G. , & Ogawa , S. ( 2012 ). Biophysical and physiological origins of blood oxygenation level-dependent fMRI signals . Journal of Cerebral Blood Flow and Metabolism , 32 ( 7 ), 1188 – 1206 . 10.1038/jcbfm.2012.23 22395207 PMC3390806

[b41] Krüger , G. , & Glover , G. H. ( 2001 ). Physiological noise in oxygenation-sensitive magnetic resonance imaging . Magnetic Resonance in Medicine , 46 ( 4 ), 631 – 637 . 10.1002/mrm.1240 11590638

[b42] Lin , A. L. , Qin , Q. , Zhao , X. , & Duong , T. Q. ( 2012 ). Blood longitudinal (T 1) and transverse (T 2) relaxation time constants at 11.7 Tesla . Magnetic Resonance Materials in Physics, Biology and Medicine , 25 ( 3 ), 245 – 249 . 10.1007/s10334-011-0287-2 PMC3752414 22071580

[b43] Lu , H. , Patel , S. , Luo , F. , Li , S. J. , Hillard , C. J. , Ward , B. D. , & Hyde , J. S. ( 2004 ). Spatial correlations of laminar BOLD and CBV responses to rat whisker stimulation with neuronal activity localized by Fos expression . Magnetic Resonance in Medicine , 52 ( 5 ), 1060 – 1068 . 10.1002/mrm.20265 15508149

[b44] Mandeville , J. B. , Marota , J. J. A. , Ayata , C. , Moskowitz , M. A. , Weisskoff , R. M. , & Rosen , B. R. ( 1999 ). MRI measurement of the temporal evolution of relative CMRO2 during rat forepaw stimulation . Magnetic Resonance in Medicine , 42 ( 5 ), 944 – 951 . 10.1002/(SICI)1522-2594(199911)42:5<944::AID-MRM15>3.0.CO;2-W 10542354

[b45] Mansfield , P. , & Maudsley , A. A. A. ( 1976 ). Line scan proton spin imaging in biological structures by NMR . Physics in Medicine & Biology , 21 ( 5 ), 847 – 852 . 10.1088/0031-9155/21/5/013 967931

[b46] Mansfield , P. , Maudsley , A. A. , & Baines , T. ( 1976 ). Fast scan proton density imaging by NMR . Journal of Physics E-Scientific Instruments , 9 ( 4 ), 271 – 278 . 10.1088/0022-3735/9/4/011

[b47] Mckeown , M. J. , Hansen , L. K. , & Sejnowski , T. J. ( 2003 ). Independent component analysis of functional MRI: What is signal and what is noise ? Current Opinion in Neurobiology , 13 ( 5 ), 620 – 629 . 10.1016/j.conb.2003.09.012 14630228 PMC2925426

[b48] Morgan , A. T. , Nothnagel , N. , Petro , Lucy. S. , Goense , J. , & Muckli , L. ( 2020 ). High-resolution line-scanning reveals distinct visual response properties across human cortical layers . bioRxiv , 2020.06.30.179762. 10.1101/2020.06.30.179762

[b49] Norris , D. G. ( 2012 ). Spin-echo fMRI: The poor relation ? NeuroImage , 62 ( 2 ), 1109 – 1115 . 10.1016/j.neuroimage.2012.01.003 22245351

[b50] Nunes , D. , Gil , R. , & Shemesh , N. ( 2021 ). A rapid-onset diffusion functional MRI signal reflects neuromorphological coupling dynamics . Neuroimage , 231 , 117862 . 10.1016/j.neuroimage.2021.117862 33592243

[b51] Oshio , K. , & Feinberg , D. A. ( 1991 ). GRASE (gradient-and spin-echo) imaging: A novel fast MRI technique . Magnetic Resonance in Medicine , 20 ( 2 ), 344 – 349 . 10.1002/mrm.1910200219 1775061

[b52] Pfaffenrot , V. , Voelker , M. N. , Kashyap , S. , & Koopmans , P. J. ( 2021 ). Laminar fMRI using T2-prepared multi-echo FLASH . NeuroImage , 236 . 10.1016/j.neuroimage.2021.118163 34023449

[b53] Pohmann , R. , Shajan , G. , & Balla , D. Z. ( 2011 ). Contrast at high field: Relaxation times, magnetization transfer and phase in the rat brain at 16.4 T . Magnetic Resonance in Medicine , 66 ( 6 ), 1572 – 1581 . 10.1002/mrm.22949 21671265

[b54] Polimeni , J. R. , Fischl , B. , Greve , D. N. , & Wald , L. L. ( 2010 ). Laminar analysis of 7T BOLD using an imposed spatial activation pattern in human V1 . NeuroImage , 52 ( 4 ), 1334 – 1346 . 10.1016/j.neuroimage.2010.05.005 20460157 PMC3130346

[b55] Ragot , D. M. , & Chen , J. J. ( 2019 ). Characterizing contrast origins and noise contribution in spin-echo EPI BOLD at 3 T . Magnetic Resonance Imaging , 57 , 328 – 336 . 10.1016/j.mri.2018.11.005 30439514

[b56] Raimondo , L. , Heij , J. , Knapen , T. , Dumoulin , S. O. , van der Zwaag , W. , & Siero , J. C. W. ( 2023 ). Towards functional spin-echo BOLD line-scanning in humans at 7T . Magnetic Resonance Materials in Physics, Biology and Medicine . 10.1007/s10334-022-01059-7 PMC10140128 36625959

[b57] Raimondo , L. , Knapen , T. , Oliveira , I. A. F. , Yu , X. , Dumoulin , S. O. , van der Zwaag , W. , & Siero , J. C. W. ( 2021 ). A line through the brain: Implementation of human line-scanning at 7T for ultra-high spatiotemporal resolution fMRI . Journal of Cerebral Blood Flow and Metabolism , 41 ( 11 ), 2831 – 2843 . 10.1177/0271678x211037266 34415208 PMC8756483

[b58] Raimondo , L. , Priovoulos , N. , Passarinho , C. , Heij , J. , Knapen , T. , Dumoulin , S. O. , Siero , J. C. W. , & van der Zwaag , W. ( 2023 ). Robust high spatio-temporal line-scanning fMRI in humans at 7T using multi-echo readouts, denoising and prospective motion correction . Journal of Neuroscience Methods , 384 . 10.1016/j.jneumeth.2022.109746 36403778

[b59] Roemer , P. B. , Edelstein , W. A. , Hayes , C. E. , Souza , S. P. , & Mueller , M. ( 1990 ). The NMR phased array . Magnetic Resonance in Medicine , 16 ( 2 ), 192 – 225 . 10.1002/mrm.1910160203 . 2266841

[b60] Sakurai , K. , Fujita , N. , Harada , K. , Kim , S. W. , Nakanishi , K. , & Kozuka , T. ( 1992 ). Magnetic susceptibility artifact in spin-echo MR imaging of the pituitary gland . AJNR Am J Neuroradiol , 13 ( 5 ), 1301 – 1308 . PMID: 1414819; PMCID: PMC8335210. 1414819 PMC8335210

[b61] Sharoh , D. , van Mourik , T. , Bains , L. J. , Segaert , K. , Weber , K. , Hagoort , P. , & Norris , D. G. ( 2019 ). Laminar specific fMRI reveals directed interactions in distributed networks during language processing . Proceedings of the National Academy of Sciences of the United States of America , 116 ( 42 ), 21185 – 21190 . 10.1073/pnas.1907858116 31570628 PMC6800353

[b62] Shen , Q. , & Duong , T. ( 2016 ). Magnetic resonance imaging of cerebral blood flow in animal stroke models . Brain Circulation , 2 ( 1 ), 20 . 10.4103/2394-8108.178544 26998527 PMC4797655

[b63] Siero , J. C. W. , Ramsey , N. F. , Hoogduin , H. , Klomp , D. W. J. , Luijten , P. R. , & Petridou , N. ( 2013 ). BOLD specificity and dynamics evaluated in humans at 7 T: Comparing gradient-echo and spin-echo hemodynamic responses . PLoS One , 8 ( 1 ). 10.1371/journal.pone.0054560 PMC3546000 23336008

[b64] Silva , A. C. , & Koretsky , A. P. ( 2002 ). Laminar specificity of functional MRI onset times during somatosensory stimulation in rat . Proceedings of the National Academy of Sciences of the United States of America , 99 ( 23 ), 15182 – 15187 . 10.1073/pnas.222561899 12407177 PMC137564

[b65] Tan Toi , P. , Jae Jang , H. , Min , K. , Kim , S.-P. , Lee , S.-K. , Lee , J. , Kwag , J. , & Park , J.-Y. ( 2022 ). In vivo direct imaging of neuronal activity at high temporospatial resolution . Science , 378 ( 6616 ), 160 – 168 . 10.1126/science.abh4340 36227975

[b66] Uludaǧ , K. , Müller-Bierl , B. , & Uǧurbil , K. ( 2009 ). An integrative model for neuronal activity-induced signal changes for gradient and spin echo functional imaging . NeuroImage , 48 ( 1 ), 150 – 165 . 10.1016/j.neuroimage.2009.05.051 19481163

[b67] Vizioli , L. , Moeller , S. , Dowdle , L. , Akçakaya , M. , De Martino , F. , Yacoub , E. , & Uğurbil , K. ( 2021 ). Lowering the thermal noise barrier in functional brain mapping with magnetic resonance imaging . Nature Communications , 12 ( 1 ). 10.1038/s41467-021-25431-8 PMC8405721 34462435

[b68] Weber , B. , Keller , A. L. , Reichold , J. , & Logothetis , N. K. ( 2008 ). The microvascular system of the striate and extrastriate visual cortex of the macaque . Cerebral Cortex , 18 ( 10 ), 2318 – 2330 . 10.1093/cercor/bhm259 18222935

[b69] Weisskoff , R. , Zuo , C. S. , Boxerman , J. L. , & Rosen , B. R. ( 1994 ). Microscopic susceptibility variation and transverse relaxation: Theory and experiment . Magnetic Resonance in Medicine , 31 ( 6 ), 601 – 610 . 10.1002/mrm.1910310605 8057812

[b70] Yacoub , E. , Duong , T. Q. , Van De Moortele , P. F. , Lindquist , M. , Adriany , G. , Kim , S. G. , Uǧurbil , K. , & Hu , X. ( 2003 ). Spin-echo fMRI in humans using high spatial resolutions and high magnetic fields . Magnetic Resonance in Medicine , 49 ( 4 ), 655 – 664 . 10.1002/mrm.10433 12652536

[b71] Yacoub , E. , Van De Moortele , P. F. , Shmuel , A. , & Uǧurbil , K. ( 2005 ). Signal and noise characteristics of Hahn SE and GE BOLD fMRI at 7 T in humans . NeuroImage , 24 ( 3 ), 738 – 750 . 10.1016/j.neuroimage.2004.09.002 15652309

[b72] Yu , X. , Qian , C. Q. , Chen , D. Y. , Dodd , S. J. , & Koretsky , A. P. ( 2014 ). Deciphering laminar-specific neural inputs with line-scanning fMRI . Nature Methods , 11 ( 1 ), 55 - 58 . 10.1038/Nmeth.2730 24240320 PMC4276040

[b73] Yu , Y. , Huber , L. , Yang , J. , Jangraw , D. C. , Handwerker , D. A. , Molfese , P. J. , Chen , G. , Ejima , Y. , Wu , J. , & Bandettini , P. A. ( 2019 ). Layer-specific activation of sensory input and predictive feedback in the human primary somatosensory cortex . Science Advances , 5 ( 5 ), eaav9053 . 10.1126/sciadv.aav9053 31106273 PMC6520017

[b74] Zhao , F. , Wang , P. , Hendrich , K. , Ugurbil , K. , & Kim , S. G. ( 2006 ). Cortical layer-dependent BOLD and CBV responses measured by spin-echo and gradient-echo fMRI: Insights into hemodynamic regulation . NeuroImage , 30 ( 4 ), 1149 – 1160 . 10.1016/j.neuroimage.2005.11.013 16414284

[b75] Zhao , F. , Wang , P. , & Kim , S. G. ( 2004 ). Cortical depth-dependent gradient-echo and spin-echo BOLD fMRI at 9.4T . Magnetic Resonance in Medicine , 51 ( 3 ), 518 – 524 . 10.1002/mrm.10720 15004793

