# Supplementary material for: Alpha-180 spin-echo-based line-scanning method for high-resolution laminar-specific fMRI in animals
Source: Imaging Neurosci (Camb). 2024 Mar 28;2:imag-2-00120. doi: 10.1162/imag_a_00120 (PMC12247619; doi:10.1162/imag_a_00120)
Supplement: Supplementary Material [file imag_a_00120-supp.pdf]

Supplementary information

Title:

Alpha-180 spin-echo based line-scanning method for high resolution laminar-specific fMRI in animals

Authors:

Sangcheon Choi[1], David Hike[1], Rolf Pohmann[2], Nikolai Avdievich[2], Lidia Gomez-Cid[1], Weitao Man[1], Klaus Scheffler[2,3], Xin Yu[1]

Affiliations:

1 Athinoula A. Martinos Center for Biomedical Imaging, Department of Radiology, Harvard Medical School, Massachusetts General Hospital, Charlestown, Massachusetts, USA

2 Max Planck Institute for Biological Cybernetics, Tuebingen, Baden-Wuerttemberg, Germany

3 Department of Biomedical Magnetic Resonance, University of Tuebingen, Tuebingen, Baden-Wuerttemberg, Germany

Supplementary Figure: 3

***Lead corresponding author:**

Dr. Xin Yu
Email: xyu9@mgh.harvard.edu
Address: 13th Street, Charlestown, MA 02129, USA

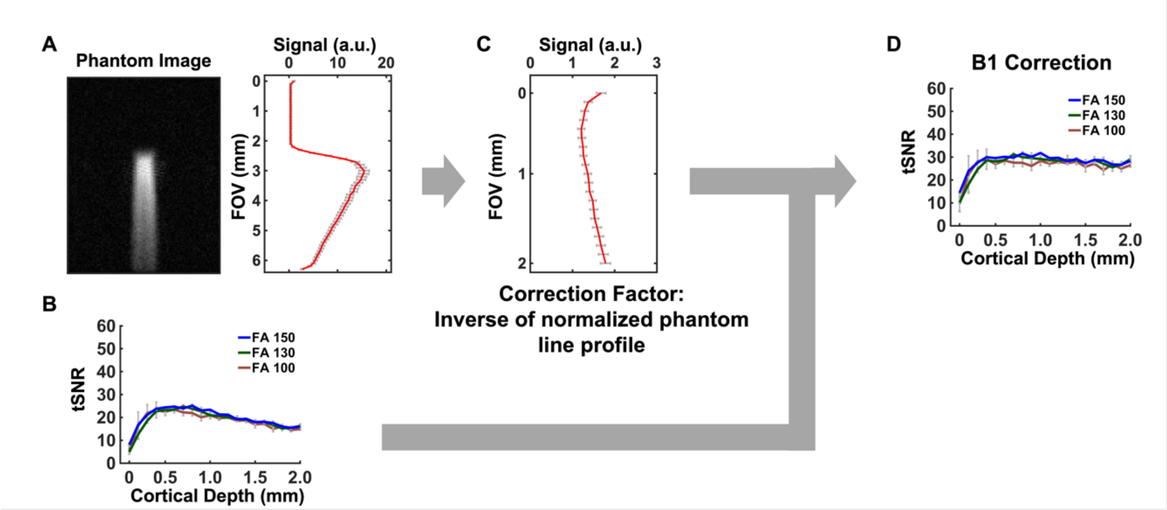

Figure S1. B1- inhomogeneity correction. **A**. Homogeneous water phnatom image with two saturation slices (left) and the line profile with phase-encoding gradient off (right). **B**. tSNR profiles of SELINE raw data (resolution: 50 µm, 1000 ms) with three excitation flip angles across the cortical depths (0-2 mm): FA 100° (5 trials), FA 130° (3 trials), and FA 150° (3 trials). **C**. Inverse of the normalized phantom line-profile (correction factor). **D**. B1 corrected tSNR as a result dividing the tSNR profiles by the normalized phantom line profile (**Fig. 3G**). Error bars represent mean ± SD.

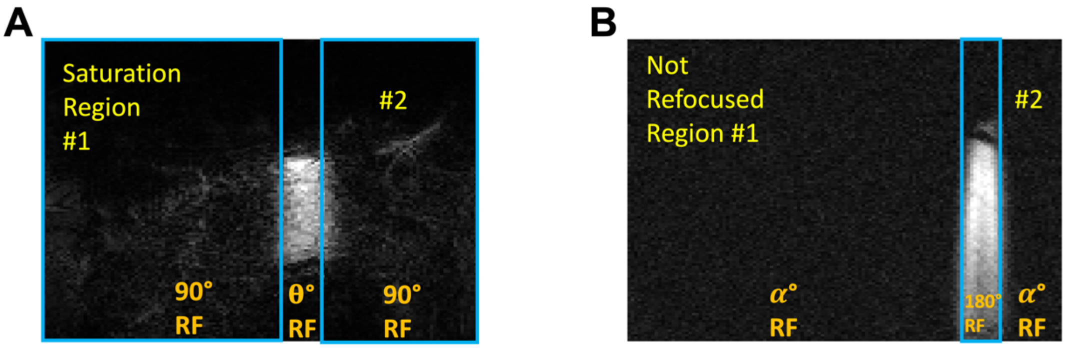

Figure S2. Comparison of GELINE and SELINE 2D images with reduced FOV. **A.** Average GELINE 2D image with outer volume suppression (TR/TE 100/12.5 ms, 3 trials). **B**. Average SELINE 2D image with inner volume selection (TR/TE 200/10 ms, 3 trials).

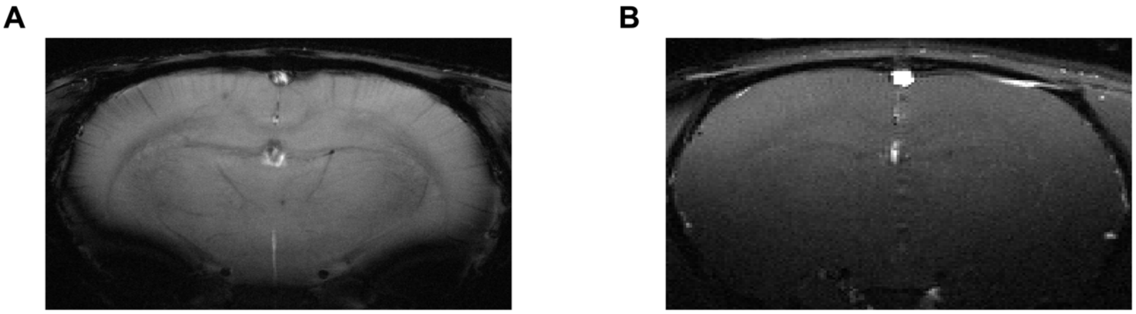

Figure S3. Comparison of GELINE and SELINE 2D images with full FOV. **A.** Average GELINE 2D image without outer volume suppression (TR/TE 100/12.5 ms, 4 trials). **B**. High temporal SELINE 2D image without inner volume selection (TR/TE 200/10 ms, 2 trials). Background images outside the ROI were cropped for clear visualization.
